# Motivations for participating in a non-interventional gender-based violence survey in a low-income setting in South Africa

**DOI:** 10.1186/s12889-017-4525-z

**Published:** 2017-06-29

**Authors:** Yandisa Sikweyiya, Mzikazi Nduna, Nwabisa Shai, Rachel Jewkes

**Affiliations:** 1 Gender and Health Research Unit, Medical Research Council, No 1 Soutpansberg Road, Pretoria, South Africa; 2 School of Public Health, University of the Witwatersrand, Johannesburg, South Africa; 3 School of Psychology, University of the Witwatersrand, Johannesburg, South Africa

**Keywords:** Gender-based violence, Motivations, Research participation, Low-income setting, Research ethics, South Africa

## Abstract

**Background:**

Qualitative study of motivations to participate in research into violence and other sensitive issues can help interpretation of findings from community based quantitative surveys. It is equally important to conduct research that may enable a deeper understanding on what motivates people to participate in GBV studies. To date, not much research has been conducted to investigate the factors that influence non-enrolment and enrolment in GBV studies from the viewpoint of the real participants. The present study sought to explore people’s reasons for participating in a non-intervention GBV community-based survey in Gauteng province, South Africa.

**Methods:**

Twenty-two qualitative in-depth interviews were conducted with adult black African men and women who had participated in a gender-based violence survey conducted in a low-income setting in South Africa.

**Results:**

Some participants reported motives for survey participation which could be interpreted as altruistic. Their motives included a desire to contribute to advancement of knowledge and to share life experiences so that unknown others could learn from these experiences. Yet, some participants hoped their participation will result in personal benefit or that they may be helped with their socio-economic challenges. The analysis further revealed a complex relationship between altruism and self-interest motives for participating in the survey amongst some of the participants.

**Conclusion:**

We conclude that it is difficult to discern which motive was primary or preceded the other. This is because such motives are not fixed, probably multiple and owing to their fluidity, may shift in people’s minds at different times and depending on the nature of the conversation. Moreover, there may be a shift in the weight given to different motives over time.

## Background

Gender-based violence (GBV) studies are considered sensitive. As such, the recruitment of participants needs to be approached with caution to safeguard confidentiality and minimise personal distress as potential participants may be victims and or perpetrators of gender-based violence. As GBV is becoming increasingly frowned upon and criminalized in many countries; research on GBV necessitates extra layers of ethical safeguards for both the researcher and the researched. However, the stringent safeguards required by research ethics committees (REC) may dissuade some scientists from undertaking GBV research for worry that RECs often consider GBV research as excessively risky and are reluctant to approve these studies. Yet, victims of GBV may want to be interviewed about their experiences and promoting research on GBV is useful for advocacy; whilst avoiding this research due to ethical clearance hurdles may relegate the issue of violence against women from national and international agendas.

Empirical data from published studies suggest that there is an array of factors that influence people to enrol in research [1]. People give varying reasons why they may enrol or not in research in the complexity of contexts that exist within and across countries around the world. For example, some authors argue that participants often are motivated to participate in research by a strong desire and willingness to help other people who have or may have the same health condition they have and to contribute towards furthering medical knowledge or research [2]. Other studies have shown that people may also be motivated by a desire to benefit family, friends or future generations, to obtain personal information or increase understanding about the result of a research experiment, condition or drug, and to do something worthwhile for interventions which may aid in curbing diseases prevalent in their communities [1, 3–9].

The limitation of many of these studies is that the overlap between self-interest and altruistic motives for research participation is often not unpacked and discussed in order to gain a better understanding of people’s reasons for research participation and the implication of this for research involving human subjects [8]. As such, not exploring, in-depth, the complexity around people’s reasons for research participation has led to a limited understanding of this issue [8, 9], and this points to the need for more targeted research to explore these issues further. This understanding is useful for ethics boards and researchers alike.

Almost all of the studies cited above were done from a clinical trials perspective; as such we know less about motivations for participation in non-intervention studies. Some authors have specifically emphasised the significance of investigating the barriers and motivations of women to participate in gender-focused studies [9, 10]. Yet, to date, there is still little scholarly focus on women’s motivations for participating in violence against women studies and what are their experiences of this [10, 11].

More research from a social science perspective on people’s motives for participating in non-intervention GBV research may not merely help develop strategies to improve the recruitment and retention of participants but also help inform consent sessions where the motivations and expectations of potential participants are explored. Furthermore, such research may help broaden our knowledge and understanding of ethics of involving women and men in GBV research [11]. The present study aimed to explore people’s reasons for participating in a non-intervention GBV community-based survey in Gauteng province, South Africa. The findings of this study are an important contribution to the literature as the study explored research participation motives of adult by men and women from a low-income setting in South Africa.

### Study context

The data presented in this article are drawn from a descriptive qualitative study that was conducted among a sub-sample of participants from a larger survey that aimed to investigate the prevalence of GBV in the Gauteng province of South Africa (see [12]). The survey was a collaborative project between GenderLinks (GL) - a non-governmental organization, the South African Medical Research Council, and the University of the Witwatersrand. Data in the survey were collected using a structured questionnaire with adult women and men in 75 randomly sampled enumeration areas (EAs) in the province. The survey fieldworkers “reached 96% of the selected households and found 89% of those had an eligible household member. Among those selected for interview, there was a 7.7% refusal rate. The overall response rate among enumerated and eligible women and men was 75%, which was 77.2% for men and 73% for women” [12].

To conduct the qualitative study, two EAs (from the 75 EAs) were conveniently selected. The two EAs were chosen because of their proximity to the South African Medical Research Council offices, where some of the authors work. These two EA’s (Thate Block and Siyakhula Extension; pseudonyms) were located in Soshanguve Township in the Gauteng Province.

The Thate Block was a relatively established section of the township. It was primarily a low-income area with few middle-class families. Siyakhula Extension was a fairly new residential area which had initially been a squatter camp—mainly a poor area with a number of shack dwellings built of corrugated iron. These two sections (blocks) were approximately 5 km apart.

In-depth interviews were used to collect the data. Interviews were conducted by YS (first author) and NS (third author). Before conducting these interviews YS had rented a room in the Thate Block and stayed fulltime for approximately three months (March to May in 2010) and was known to the community to be a researcher.

## Methods

### Participants and procedures

The data analysed for this article results from 22 in-depth interviews, 10 conducted with women and 12 with men. The larger survey randomly selected 20 households per EA for interview. Potential participants for the survey were told that participation was voluntary, and that there will not be direct benefit for them in participating in the survey. Furthermore, they were informed that the interview would be anonymous and that data will be kept in strict confidence. All women were provided information on how to access services that help women experiencing abuse. After giving written informed consent, one eligible man or woman was systematically selected from those who slept four nights a week or more in the household. This yielded a sample of 511 women and 487 men for the survey [12]. The fieldworkers managed to interview 12 men in the Thathe Block and 12 women in Siyakhula Extension.

Before the commencement of survey in these two EAs, YS requested the fieldworkers to invite the survey participants for the qualitative study and all 24 participants agreed to be contacted for the qualitative study. Initially, they were contacted telephonically and thereafter met face to face for interviews, two weeks to a month after they had completed the survey. Eleven men were interviewed by YS and 10 females were interviewed by NS, a female researcher. Two females and one male could not be located for interview after several attempts. One man was interviewed twice after he requested another interview as he felt he had been dishonest in the first interview. The information he shared in the first and second interviews, after being verified for consistency and reliability, was included in the analysis. Participants’ age ranged between 22 and 67 years, with the majority (*N* = 15) over 50. Eight were married, three were single, seven were dating and two were cohabiting. Most participants (*N* = 16) were not formally employed at the time of the interview. Interviews with men were conducted in isiZulu and those with women were a mixture of isiZulu and seTswana.

A semi-structured interview guide was employed to conduct the interviews which were audio-recorded. Initially, the interview guide comprised only a few broad questions, with possible probes drafted. Participants were asked about their experiences of the survey including their reasons for participating and how it had impacted them, how answering the sensitive questions had made them feel, whether the research, directly or indirectly, was harmful or helpful to them and how, and whether they experienced adverse consequences as a result of their participation in the survey. Whilst we interviewed everyone who was available and had given written informed consent, we believe that by the time we conducted the last interviews we had reached saturation as we were not obtaining new information from these interviews.

### Data analysis

To analyse the data we used thematic content analysis [13] and the analysis was led by the first author (YS). In line with the dictates of the content analysis approach, YS first repeatedly read the transcripts to familiarize himself with the data. He then coded the transcripts and identified open codes. Further to this, he grouped similar codes to form categories, a process which enabled him to inductively generate eight broad categories, which he then defined [14]. The categories were then populated with relevant text from the transcripts to ascertain whether these categories were grounded in data. Three categories which proved to be supported by data were retained in the analysis, and the rest were discarded. The retained categories were then interrogated for underlying meanings [13].

As the next stage of analysis, the co-authors (MN, NJS and RJ) joined in the interpretation of the findings. An intense discussion about the retained categories was held. The interpretation focussed on the meanings of the categories how they fitted together, what patterns, hypothesis and mini-theories were emerging [14]. Lastly we compared our findings with results of similar published studies and made conclusions.

## Results

In the interviews participants reported that their survey participation was motivated by a number of reasons from which three thematic areas were identified: altruism, self-interest, and interconnectedness between altruism and self-interest, and we present the findings of our analysis and interpretation below.

### Altruistic motives

A total of seven participants reported to have been motivated to participate in the survey by reasons which could be interpreted as altruistic. Specifically, they mentioned that they were motivated by the desire to share their lived experiences in order to contribute to knowledge generation and for unspecified others to learn from such experiences.

#### Sharing own experiences to contribute to advancement of knowledge

Amongst the participants who reported altruistic motives, most said that their participation in the survey and act of sharing personal information was intended to help the researchers answer the research questions, thereby contributing to knowledge generation. This motive is best illustrated by the following accounts from Mapaseka: “I sometimes think whether the findings will be read as a book, given to women to read as a book, women who are coming after us will read these terrible things we experienced” (A woman aged 64). Here the participant hoped that the data would be accessible and useful to others, in particular women in similar circumstances as them. This highlights a sense of the valuing of intergenerational transmission of knowledge and a commitment to use personal stories and experiences to advance change.

Mapaseka further expressed that:

So the person I told my experiences does not stay here, maybe she is going to write about it, write it into a book and give it to other women. There are women coming after us, they are still growing…the book that will be produced will teach them that there was once a woman that experienced things like this; she had a difficult life, struggling until she succeeded and got a shack and stayed with her child. (Mapaseka, a woman aged 64)

Sharing struggles and triumphs seemed central in Mapaseka’s motivations. The encounter with a field worker who was a stranger appear to have made the sharing easier. This suggests that the participant was thoughtful about her participation. Thought was given to what, how and to whom to share and she had her audience in mind. This is important as it suggests that even poorer people with little education do not blindly agree to partake in scientific studies.

In the same vein, when asked to share her thoughts on participating in the survey, Mathapelo mentioned that she believed that her participation: “…did help you know…I don’t know how to put it, but it helped, and maybe they’ll still [use it to] help others out there”. (Mathapelo, a woman aged 34). A somewhat similar view was shared by Mobutho, he posited:

Well if someone comes from a certain organization, like your organization, doing research, no matter what question you ask me, I will not have a problem because I can see, just like you, you are doing research… I can see it will help you somewhere and somehow in the future with your research. (Mobutho, a man aged 43)

An initial reading of these narratives seems to suggest that these participants had solely intended to share their personal information so that unrelated others may benefit from reading about their experiences without them expecting reciprocity or personal reward in return. Yet, as it is apparent below, these motives may have been linked to another motive(s).

### Self-interest motives

#### Participation will result in personal benefit or reward

Our analysis revealed that some participants had expectations that something beneficial will accrue for them from their participation in the survey. The following accounts describe these expectations:

Because there at the hospital when I was sick staying there, they once sent someone to help us to recover, so I thought with that research [the survey] they were trying to help us. So I thought they were here to help us, maybe it was free health care, things like that. So that is what I thought after he had left, that with the thing I did [survey participation], I will get help. (Kelebogile, a man aged 41)

It was not clear if this was immediate help or if the hope for help was still there beyond the fieldwork period. In survey studies there is no prolonged engagement with the community and hence no action for the community as a direct result of such. This is also the case with commissioned studies, and this was one of those, the research firm usually leaves for the next consultancies with no resources to support the implementation of findings or respond to participant’s other expectations and needs. A relatively similar expectation is evident in Cleopatra’s assertion to follow:

About the things that you asked me…as I said, I thought that you may have been here to help me with my boy. Beyond that I don’t know how my participation will help me or what it will bring for me. (Cleopatra, a woman aged 62)

Accentuating further the expectations held by some participants, Mobutho argued that researchers wouldn’t engage in a research study without a purpose, and thus believe that, that purpose translates to benefit for research participants and others. He posited:

Yes because I don’t think you are doing research for nothing, you are doing research to get something that will help us and even help other people. No one can do something for nothing. So you are busy doing this research in order to get something, that cure that will help people. (Mobutho, a man aged 43)

#### Support with livelihoods challenges: the need to make ends meet

The word ‘help’ strongly featured in the narratives of some participants as an expectation they had from their survey participation. It was an expectation held by several participants, and the nature of assistance expected varied. For example, while Nonhlanhla was living with HIV she felt she was well enough to work, yet due to scarcity of jobs in South Africa, especially for people with few years of formal schooling, she was unemployed and thus had hoped that through her survey participation she would be assisted to find a job. She commented:

“The only support I need is to get a job… I just want a job, I’m not sick; I have the strength, since I’m taking my medication I don’t have a problem – I can work. Everything – the rent – it’s all facing me”. (Nonhlanhla, a woman aged 49).

This study did not have a focus on employment or social security issues. Furthermore, job placement assistance was not a benefit promised nor written in the survey recruitment materials and therefore it is unlikely that it was mentioned during the informed consent process. It is thus unclear how Nonhlanhla linked her research participation to chances of being helped with a job. It is possible that it was not the study itself that was expected to facilitate this but that meeting other people (the researchers) who may have some exposure and connections to opportunities could have been perceived as a catalyst for her to access job opportunities.

Kelebogile was also living with HIV, but in contrast to Nonhlanhla, hoped he would be helped with his medical treatment for his condition. He posited:

I read the letter [participant information leaflet] very well and understood that I should speak the truth, because if I lie, then maybe I will not get help, but if I speak the truth, that is where I am going to get help. Generally I am happy but the times I think about my condition, my heart becomes painful you see. (Kelebogile, a man aged 41)

The participant information sheet and the consent forms used in the survey did not refer to any offers for help. On the contrary it stated that there would be no direct personal benefits from participating in the study. Yet, apparent in the above extract, for Kelebogile the “help” he hoped for was contingent on him being candid in his responses in the survey interview. This underscores the strength of the motive- “expectation of help”- in driving his survey participation. The extract to follow describes Sipho’s situation. In the interview he reported to be struggling financially and that he was also suffering from HIV/AIDS. Similar to Kelebogile, Sipho held out hope that through his survey participation he may be helped with food parcels or financially to alleviate poverty at home. In the narrative below Sipho explained his reason for requesting a second interview with the researcher:

It is because I was worried about this food parcel thing. So I did not know whether you were asking that information so that you can arrange food parcels for me or what, or maybe there is another form of help you were coming with… I thought I’ll see when you get here. That is why I followed it up, I was not thinking about too much. That is the only thing. (Sipho, a man aged 40)

Sipho was interviewed twice by YS. In the first interview he was evidently suspicious of the researcher and divulged little about himself. Few days later, he called YS and requested to redo the interview. He subsequently disclosed that he was living with HIV and that he was guarded in the first interview as he didn’t trust the researcher. He asserted:

So that time you first came here I decided not to tell you the truth, but in truth I am sick. There is also something that bothered me when you first interviewed me, I have a problem here, my problem is that I owe rent here in the house and I am not the reason why I’m owing rent. (Sipho, a man aged 40)

In the second interview, Sipho further reported that after the first interview he became distressed as he reasoned that by being guarded in the interview he may have forgone an opportunity to be helped, a view he said was shared by his wife. Ironically, the information that he shared in the second interview was not about the research participation but about his state of penury. He opened up about his penury, his illness and financial difficulties, he seemed to have used the (second) interview to paint a picture of himself as a needy person as if to make a point that he ‘qualifies’ for social assistance that could be arranged for participants.

#### Shared characteristics among participants with self-interest motives

Our analysis revealed that the self-interest motive- “expectations of help” -from survey participation was dominant among participants who possessed certain characteristics. For example, Nonhlahla, Kelebogile and Sipho shared somewhat similar characteristics in that they all reported to be living with HIV, unemployed and struggling financially, while Thandaza was suffering from arthritis and also reported to be poor. Our analysis shows that these participants mainly participated in the survey motivated by the hope to self-benefit. The following extracts support this interpretation.

Yes because we are sick and we are not working, so I thought that maybe we will be helped. That is why I did not have a problem the time he [survey researcher] got here and after he had left… yes I would be very happy if you would pressurize these [medical] doctors so that we can be cured, they should find the cure, so that we can be cured. That is the ultimate thing for me, every day when I sleep that is the only thing I think about. If I would wake up one day and hear that a cure has been found, I would be very happy because this thing [AIDS] is really killing us. (Kelebogile, a man aged 41)

Similarly, Mobutho and Margaret, while they did not report to be living with a long-term illness, also described their socio-economic situation as poor, and hoped that personal benefit may accrue from their survey participation. In explaining her situation at home, Margaret posited:

I told them in my home we are very poor, my husband beats me, what should I do? I’m struggling, so what then? They [researchers] did not give me a full answer. (Margaret, a woman aged 46).

The help expected was not always material but also in the form of information and advice. However, our data reveals that, in the context of limited resources, participants whose socio-economic situation was poor and had weak livelihoods may participate in research expecting self-benefit.

In our analysis, the characteristics of participants who reported self-interest motives were somewhat similar, while conspicuously dissimilar to those of participants who did not report self-interest motives. The latter group mainly comprised of young people who had tertiary education and had more knowledge about or had been previously exposed to research. As this group had a better appreciation of the research, they were likely to know what may or may not accrue from research participation.

### Interconnectedness between altruism and self-interest motives

Further analysis revealed a complex relationship between altruism and self-interest motives for participating in the survey amongst some of the participants in our sample. In the interviews, a number of participants simultaneously reported these motives. Below we present narratives that best display the interconnectedness between altruistic and self-interest motives amongst participants in this study. For example, when asked how he perceived the survey, Kelebogile posited:

That interview really made me strong; I know that I will be remembered for my contribution. I also know that there are people who think of me. And maybe one day, I will get help. That hope is still alive… (Kelebogile, a man aged 41)

Thandaza reflected on her decision to participate in the survey, she said:

So this was the first time I hear of it [research]. So I thought maybe this woman, the way she is asking, maybe she can help many other people and maybe help us too, so I accepted it [invite to participate]. (Thandaza, a woman aged 50).

Even with limited knowledge about research, this participant connected with the research assistant; was drawn into the study by the rapport that the research assistant created at recruitment. It is in response to this initial contact that the motives for participation are shaped; this can be shaped by the content, the manner of communication and framing of the study by the researcher.

She further elucidated:

I meant that I did not understand well, as I said this woman came with some questions. So I told myself that this woman is working or volunteering…she is someone who needs to do her job. Hence jobs are so scarce these days, when a person comes into your house, you should welcome him/her and answer where you can, maybe her life would progress, so as mine. (Thandaza, a woman aged 50)

The extracts above clearly demonstrate that altruistic and self-benefit motives equally influenced some participants’ decision to participate in the survey. Indeed many participants reported being motivated by the need to help unknown others, while also hoping to benefit personally or as a collective of people infected by HIV, affected by GBV and poverty. This is further evidenced by Mobutho’s narrative to follow:

Let us talk about AIDS research, these people when they do research they want to get a cure. So they need information from people in order to help them get the cure for this disease. So it is the same with your research, you need something that will help you, which will help us in the future. (Mobutho, a man aged 43)

## Discussion

This article has shown that for most participants in this study altruism and self-interest motives were behind their participation in the survey [15]. It was not always possible to discern these as they sometimes coexisted. This finding is analogous to that reported by Godskesen et al. [16] where they reported that “hope for cure” and “altruism” were the main motives for people to participate in phase 3 clinical cancer trials in Sweden. In our study, a number of participants were content with the realization that they will be recognised for their contribution, yet simultaneously held out hope that their contribution will also yield personal benefit [1, 7, 17]. However, it is difficult to discern which motive was primary or preceded the other.

Furthermore, explicating the complex interconnectedness between altruism and self-interest motives amongst research participants is particularly challenging. Indeed, in many published studies altruism and self-interest motives have been reported as reasons for people to participate in research [8, 18, 19]. For example, findings of a systematic review of studies published in the United States showed that people’s motivations for research participation included the desire to help their family or community, advancing medical knowledge, monetary incentive, free lunch or free health examination [15].

However, the weakness of the current analysis in many of these studies is that authors often suggest that motives for research participation operate as discrete categories and have a fixed hierarchy (e.g. primary and secondary motives). Our analysis reveals that in real life this may not always be the case.

In this article, we have shown the interlocked and muddled nature of the motives shared by the participants in this study. And findings of the current study offer insights into the intricate decision making processes research participants undertake when enrolling in studies [20]. To accentuate this further, Garcia-Moreno et al. report of a female participant interviewed in Peru in the WHO multi-country study on violence against women as having reflected on her survey participation as follows: “I feel very good because I believe it will help many women knowing about these things, and even if this help will not reach me, I know it will reach many women” [21]. This participant’s account suggests that she was motivated by the desire to help unknown women, yet arguably it also seems, to a certain extent, she may have hoped to benefit personally from her participation in the survey. As such we argue that people may often agree to research participation without giving too much thought as to why they are agreeing to enrol in the study and the future implications of their decision [22].

We have also demonstrated that people’ motives for research participation are extremely difficult to untangle as such motives are not fixed and probably multiple [23]. People may be motivated by varying things at different times, as such it is essential to recognise that reasons for enrolling in studies may be numerous and nebulous [24]. Furthermore, there is need to recognise that research participation motives may shift in people’s minds at different times and depending on the nature of the conversation. Likewise, there may be a shift in the weight given to different motivations over time. For example, immediately after research participation an explanation that “I don’t know why I agreed to participate” may not be often perceived by researchers as a good enough explanation, or research participants may be uncomfortable to provide such an answer immediately after having been taken through a study information giving session with the researcher, and therefore may be inclined to provide a socially desirable answer [25]. On the other hand, it is essential to note that, as Schaefer and co-workers [23] argue, the process of decision making for enrolling in research is a dynamic one that is reviewed continually.

In this article, we have demonstrated that most of the participants who hoped to self-benefit from survey participation also reported to have weak livelihoods and that they were struggling financially. This finding reflects other studies. For example, in her paper Viens queries: “how the combination of lower levels of education and higher levels of economic need impact informed consent with people with depressed socio-economic backgrounds?” [19]. Our findings suggest that people’s poor socio-economic circumstances may play a critical role on their decision to participate in research as they tend to do so carrying hopes to benefit materially from the research. This finding receives support from studies conducted in resource-poor settings in Africa [26, 27]. However, this finding should be interpreted with caution as Sharp et al. [9] argue that the evidence that participation in research is modified by socio-economic circumstances is currently scanty and that more research is needed to demonstrate this. On the other hand, Appelbaum et al. [28] argue that people in need may have deep-seated and unwavering hope that their research participation will positively affect their lives. Furthermore, it is known that people in adverse circumstances of various forms often have fantasies of rescue from these harsh circumstances. As such, in the present study, some people may have been clutching on these fantasies of a better life, or change in their circumstances, accruing from survey participation as such fantasies provide hope and thus serve to sustain people in adversity. For example, Kelebogile’s hope to wake up one day and be told that a cure for HIV has been found may be a hope, or fantasy, that enabled him to continue having a positive outlook in life in the face of considerable adversity.

### Limitations

The coding of the data was done by a single researcher (YS), and this may have introduced bias in the analysis presented here. However, to enhance the rigour of the analysis, YS continuously presented his emerging code book to RJ, who has extensive expertise in analysing qualitative data, for discussion and verification. Notwithstanding this, it is recommended that two or more people be involved in data coding in order to enhance the quality of the analysis through attaining a high inter-rater agreement between those conducting the coding.

For our sample, we do not think that there was selection bias as all 24 participants who completed the survey and were approached to participate in the qualitative interviews agreed to participate. Second, those we could not interview were either unavailable due to work related commitments during the data collection period or could not be reached on their phones.

## Conclusions

The findings of this study have important implications for ethics review of non-intervention studies involving human participants. Specifically, the finding that motives for research participation appear to be multiple and not easy to untangle may have some bearing on informed consent. As such, the process of informed consent needs to take into account the intertwined nature of motives and for researchers to be cognisant of these as people enrol and participate in research studies.

We have presented findings that show that a number of participants expected something in return for their participation in the survey. As such, we recommend that RECs should pay more attention to the language of the recruitment methods and to encourage researchers to guard against participants’ misconceptions or false expectations of direct benefit in non-intervention studies. In line with this, we argue that it would be helpful for RECs to encourage researchers to spend adequate time discussing with participants about their expectations for their participation in research during the initial informed consent stage [29].

Based on the findings of this study, we suggest that research ethics committees could view the issue of research induced hope in two ways. First, RECs could view this as research participation that is occurring under false expectations which is incompatible with informed research consent (even if researchers had not done anything to fuel these expectations). Alternatively, RECs may view this as a situation where the research provided an opportunity for hope for better life circumstances through something good resulting from research participation, which enabled the participant to get through another day with slightly elevated hope. We argue that the latter scenario may be perceived as a very good thing, a direct psychological benefit for the research participant rather than a problematic misconception.
